# A pyroptosis‐related signature predicts prognosis and indicates immune microenvironment infiltration in glioma

**DOI:** 10.1002/cam4.5247

**Published:** 2022-09-26

**Authors:** Jia Chen, Shanwei Chen, Bingxian Li, Shaojiong Zhou, Han Lin

**Affiliations:** ^1^ The Fourth People's Hospital of Chengdu Chengdu China; ^2^ The Clinical Hospital of Chengdu Brain Science Institute MOE Key Lab for Neuroinformation, University of Electronic Science and Technology of China Chengdu China; ^3^ Department of Neurosurgery, Guangdong Provincial People's Hospital, Guangdong Academy of Medical Sciences Guangzhou China; ^4^ Shantou University Medical College Shantou China; ^5^ Department of Neurology, Shantou Central Hospital Shantou China; ^6^ Department of Neurosurgery, Beijing Tiantan Hospital Capital Medical University Beijing China

**Keywords:** glioma, immune checkpoint, immune profile, immunotherapy, pyroptosis

## Abstract

**Background:**

Glioma, the most common malignant brain tumor, leads to high recurrence rates and disabilities in patients. Pyroptosis is an inflammasomes‐induced programmed cell death in response to infection or chemotherapy. However, the role of pyroptosis in glioma has not yet been elucidated.

**Methods:**

RNA‐seq data and clinical information of 660 gliomas and 847 samples were downloaded from the TCGA and CGGA, respectively. Then, data of 104 normal brain tissues was retrieved from the GTEx for differential expression analysis. Twelve pairs of peritumoral tissue and glioma samples were used for validation. Gene alteration status of differentially expressed pyroptosis‐related regulators in gliomas was detected in cBioPortal algorithm. Consensus clustering was employed to classify gliomas based on differentially expressed pyroptosis‐related regulators. Subsequently, a PS‐signature was constructed using LASSO‐congressional analysis for clinical application. The immune infiltration of glioma microenvironment (TME) was explored using ESTIMATE, CIBERSORT, and the other immune signatures.

**Results:**

cBioPortal algorithm revealed alteration of these regulators was correlated to better prognosis of gliomas. Then, our study showed that pyroptosis‐related regulators can be used to sort out patients into two clusters with distinct prognostic outcome and immune status. Moreover, a PS‐signature for predicting the prognosis of glioma patients was developed based on the identified subtypes. The high PS‐score group showed more abundant inflammatory cell infiltration and stronger immune response, but with poorer prognosis of gliomas.

**Conclusion:**

The findings of this study provide a therapeutic basis for future research on pyroptosis and unravel the relationship between pyroptosis and glioma prognosis. The risk signature can be utilized as a prognostic biomarker for glioma.

## 1 INTRODUCTION

Glioma, the most common malignant brain tumor, is histologically characterized by considerable cellularity and mitotic activity, vascular proliferation, and necrosis.^1^ Glioma patients suffer from high recurrence rates and disabilities due to tumor invasiveness and chemotherapy and radiotherapy resistance.^2^ Currently, tumor resection, adjunctive chemotherapy, and radiotherapy are the standard means for glioma therapy.^3^ However, these treatment options have limited therapeutic effects on glioma; thus, the disease remains a serious clinical problem.^4^ This calls for elucidation of glioma tumorigenesis and exploration of glioma biomarkers, with the overall goal of developing effective therapeutic strategies.

Pyroptosis, an inflammasome‐induced programmed cell death mediated by gasdermins, is thought to modulate clearance of pathogens from infections and represents caspase dependence, nuclear condensation, and DNA fragmentation.^5^, ^6^ However, unlike apoptosis, it is critically dependent on plasma membrane pores formed by members of the gasdermin protein family, which is usually the consequence of inflammatory caspase activation.^7^ Although the release of inflammatory factors in pyroptosis might create a tumor‐suppressive environment, pyroptosis can hold multiple roles in various cancers. Production of pyroptosis‐related regulator and inflammasomes has been shown to suppress tumor growth (colorectal cancer, liver cancer, and nasopharyngeal carcinoma), whereas pyroptosis‐related inflammasome might promote the growth of cancer cells in melanoma.^8^, ^9^, ^10^, ^11^ Some studies have revealed that several regulatory noncoding RNA could induce pyroptosis in glioma tumor cells and inhibit their proliferation in vitro.^12^ However, the inflammatory microenvironment created by pyroptosis played a significant role in the invasion and growth of glioma.^13^, ^14^ Zhang et al. newly identified a four pyroptosis‐related gene signature (CASP4, CASP9, GSDMC, and IL1A) which had an impact on the TME and immune cell infiltrations of glioma.^15^ Besides, a risk model of 10 pyroptosis‐related genes was constructed to predict the prognosis of patients with glioma.^16^ However, comfirmatory experiments were lacking for these findings. Therefore, given the complexity of the microenvironment, studies should be conducted to thoroughly elucidate the biofunction of pyroptosis‐related regulators and their effect on glioma prognosis.

In this study, gliomas were obtained from The Cancer Genome Atlas (TCGA) database, Chinese Glioma Genome Atlas (CGGA) and The Genotype‐Tissue Expression (GTEx) Project. Then, we screened differentially expressed pyroptosis‐related regulators in gliomas and explored their potential biofunction. Moreover, this study identified two pyroptosis‐related clusters of glioma samples, which showed significantly different molecular characteristics and immune cell infiltration. Subsequently, a PS‐signature was established based on the identified subtypes, followed by exploration of the biological pathways associated with the risk signature. Notably, the PS‐signature showed the potential to be a biomarker for glioma diagnosis and reflected the immune microenvironment of gliomas.

## 2 METHOD AND MATERIAL

### 2.1 Defining pyroptosis‐related regulators and data processing

Forty‐two human pyroptosis‐related regulators were defined from five sources, including Zhang, Rogers, He, Shi, and Gene Ontology (GO) project.^17^, ^18^, ^19^, ^20^, ^21^ Next, FPKM RNA‐seq data of 694 gliomas were obtained from LGG and GBM datasets in The Cancer Genome Atlas (TCGA, https://portal.gdc.cancer.gov). From the Chinese Glioma Genome Atlas (CGGA, https://www.cgga.org.cn), we downloaded RNA sequencing data of 1018 gliomas from 693_cohort and 325_cohort. In addition, we retrieved the RNA‐seq data of 104 normal brain tissues from the Geneotype‐Tissue Expression database (GTEx, https://www.gtexportal.org/) for differential expression analysis.^22^ The “ComBat” algorithm of the “SVA” R package was employed to eliminate the batch effects between different datasets. Patients with incomplete clinicopathologic information were excluded.

### 2.2 Analysis of differentially expressed pyroptosis‐related regulators in glioma and identification of pytoptosis‐related patterns

The “limma” R package was used to obtain differentially expressed pyroptosis‐related regulators (cut‐off: adjusted (*p* < 0.05 and | log2FC) | >0.5). STRING (http://www.string‐db.org), a website tool for analyzing protein–protein interactions, was applied to visualize the network of differentially expressed pyroptosis‐related regulators and perform functional enrichment analysis.^23^ In the Human Protein Atlas (HPA) online database (Protein Atlas version 20.1, http://www.proteinatlas.org/), we compare the expression of pyroptosis‐related regulators at a translational level.^24^, ^25^, ^26^ In addition, mutation and copy‐number alteration (CNA) status of differentially expressed pyroptosis‐related regulators in gliomas was further explored in the cBioPortal algorithm (http://www.cbioportal.org/), followed by evaluating the impact of pyroptosis‐related regulators' alteration on glioma prognosis.^27^ Classifying samples by predefined gene expression characteristics has been proven to be an important method to distinguish samples for further analysis.^28^ The consensus clustering algorithm from the “ConsensusClusterPlus” R package was performed to identify cluster members in glioma on the basis of the expression of pyroptosis‐related regulators.^29^ Taking clinical data into consideration, we determined the difference in the prognosis of samples between the clusters.

### 2.3 Construction and validation of a PS‐related signature

It is well known that a sparse linear predictive model can be constructed using the Lasso algorithm.^30^ Herein, we constructed an efficient prediction model using LASSO analysis.

**PS‐score** = $\begin{array}{c} \sum_{i}^{n}\text{expression level of pyroptosis}-\text{related factors} \end{array}$ $\begin{array}{c} \left(i\right)*\beta \left(i\right) \end{array}$ (β: coefficient of key pryoptosis‐related regulator).

Kaplan–Meier survival curves were performed to compare the prognosis of different PS‐score groups. Time‐dependent ROC curves (a quantitative analysis tool for a model) were generated to determine the efficiency of the model. Signature‐related genes were integrated in the MSigDB database for functional analysis.^31^ The “rms” R‐package was used to establish a nomogram (a statistical model with a user‐friendly graphical interface) with the goal of predicting the clinical outcomes of glioma patients. Notably, a calibration plot was generated to assess the capacity of the nomogram.^32^

### 2.4 TME cell infiltration, tumor mutation burden (TMB), and stemness score of gliomas

A previous study reported that tumor‐infiltrating immune cells in the microenvironment are closely associated with the immune response to tumor pyroptosis.^33^ In this study, we used the LM22 signature from CIBERSORT and 28 previously reported immune cells signatures to quantify the proportions of immune cells in glioma.^34^ In addition, the fraction of immune and stromal cells in glioma samples was calculated using the “Estimate” method.^35^ Given that some tumoral parameters play significant roles in tumor immune response, their association with the PS‐signature deserved to be analyzed. It should be noted that the tumor mutation burden (TMB) is associated with abundance of antigens and neoantigens, which leads to increased immunogenicity.^35^, ^36^ High stemness indices (mDNAsi) showed a strong association with pathologic grade, aggressiveness, and poor clinical outcomes.^37^ Therefore, this study compared the TMB and stemness indices of different groups in gliomas.

### 2.5 RNA extraction and real‐time quantitative PCR (qRT‐PCR)

Total RNA was extracted from 12 pairs of adjacent non‐tumor brain tissues and glioma samples by means of a TRIzol reagent (AG21102, Accurate Biotechnology). Subsequently, the extracted RNA was reverse‐transcribed using the PrimeScript RTMasterMix kit (AG11706, Accurate Biotechnology). Finally, the SYBR GREEN Kit (AG11701, Accurate Biotechnology) was used to perform qRT‐PCR and quantify pyroptosis‐related regulators in gliomas and brain tissues in accordance with the manufacturer's protocol. Table 1 shows the sequences of primers used for qRT‐PCR. Notably, relative mRNA expression was normalized to *GAPDH* (housekeeping gene) mRNA expression and quantified using the 2 − ΔΔCt method.

**TABLE 1:** Primer of pryoptosis‐related regulators

Gene	Forward	Reverse
*TREM2*	5′‐AGACTACTCTGCCTGAACACT‐3′	5′‐CCAGCTAAATATGACAGTCTTGGAT‐3′
*IL18*	5′‐AGAGATAATGCACCCCGGAC‐3′	5′‐ACACTTCACAGAGATAGTTACAGCC‐3′
*CASP3*	5′‐GACTCTGGAATATCCCTGGACAACA‐3′	5′‐AGGTTTGCTGCATCGACATCTG‐3′
*TP53*	5′‐GAGGCCTTGGAACTCAAGGATG‐3′	5′‐TCAGGCCCTTCTGTCTTGAACA‐3′
*AIM2*	5′‐AAAAGCTGGTGAAACCCCGAA‐3′	5′‐CATTGTGTCCTCGTTTCTAACCC‐3′
*GSDMB*	5′‐AGGAAACCCTGAAAAGCGACC‐3′	5′‐GCACCATCCTTCTTCATCGTCT‐3′
*IL1B*	5′‐TGAAGCAGCCATGGCAGAAG‐3′	5′‐GGTCGGAGATTCGTAGCTGGA‐3′
*CASP1*	5′‐CAAGTCAAGCCGCACACGTCT‐3′	5′‐AGCTCTGTAGTCATGTCCGAAGCA‐3′
*CHMP6*	5′‐CGGCAAATAGACGAGCTCCT‐3′	5′‐CTCTATTTGTTCCTGAGTGATTGCG‐3′
*GZMA*	5′‐TATGACCCAGCCACACGCGAA‐3′	5′‐GGTTCCTGGTTTCACATCGTCCC‐3′
*GAPDH*	5′‐GCCATCACAGCAACACAGAA‐3′	5′‐ GCCATACCAGTAAGCTTGCC ‐3′

Abbreviations: *AIM2*: absent in melanoma 2; *CASP1*, caspase 1; *CASP3*: caspase 3; *CHMP6*: charged multivesicular body protein 6; *GAPDH*: glyceraldehyde‐3‐phosphate dehydrogenase; *GSDMB*: gasdermin B; *GZMA*, granzyme A; *IL18*, interleukin 18; *IL1B*: interleukin 1 beta; *TP53*: tumor protein 53; *TREM2*, triggering receptor expressed on myeloid cells 2.

### 2.6 Statistical analysis

Correlation analysis was calculated using Spearman's test. Log‐rank tests and Kaplan–Merier curve were derived to determine the prognosis differences between different groups of glioma patients. Wilcoxon test and the Kruskal–Wallis test were used to compare differences between groups. *p* < 0.05 was considered statistically significant.

## 3 RESULT

### 3.1 Expression variations and genetic changes of pyroptosis‐related regulators in glioma

**FIGURE 1:**
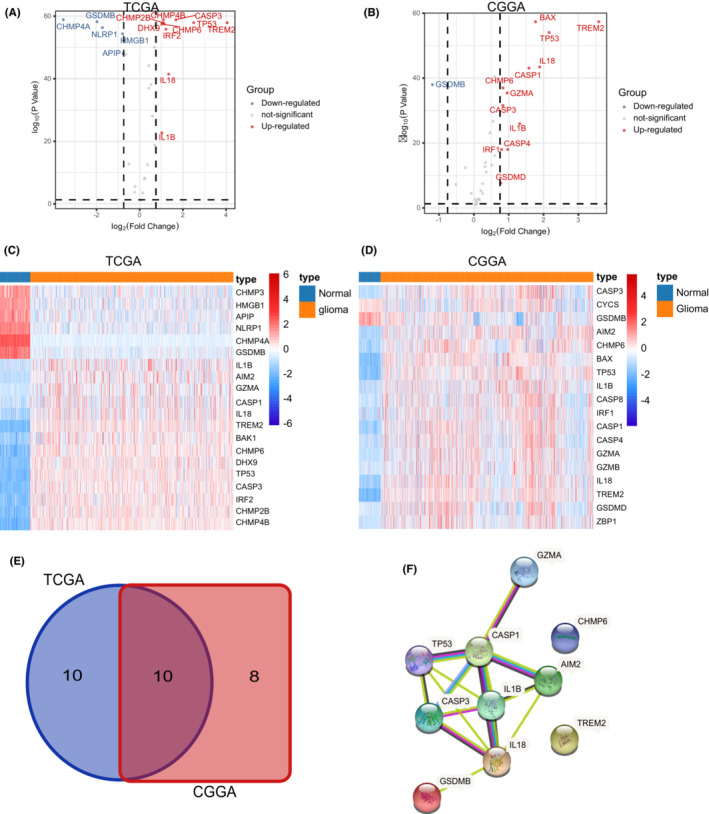
Differentially expressed pyroptosis‐related regulators between glioma and normal brain tissue. (A) Volcano plot showing the differentially expressed pyroptosis‐related regulators between gliomas and normal brain tissues in the TCGA‐GTEx cohort. (B) Volcano plot showing the differentially expressed pyroptosis‐related regulators between gliomas and normal brain tissues in the CGGA‐GTEx cohort. (C) Heatmap showing the differentially expressed pyroptosis‐related regulators between gliomas and normal brain tissues in the TCGA‐GTEx cohort. (D) Heatmap showing the differentially expressed pyroptosis‐related regulators between gliomas and normal brain tissues in the CGGA‐GTEx cohort. (E) Venn diagram showing common differentially expressed pyroptosis‐related regulators. (F) Protein interaction network (PPI) of common differentially expressed pyroptosis‐related regulators.

**FIGURE 2:**
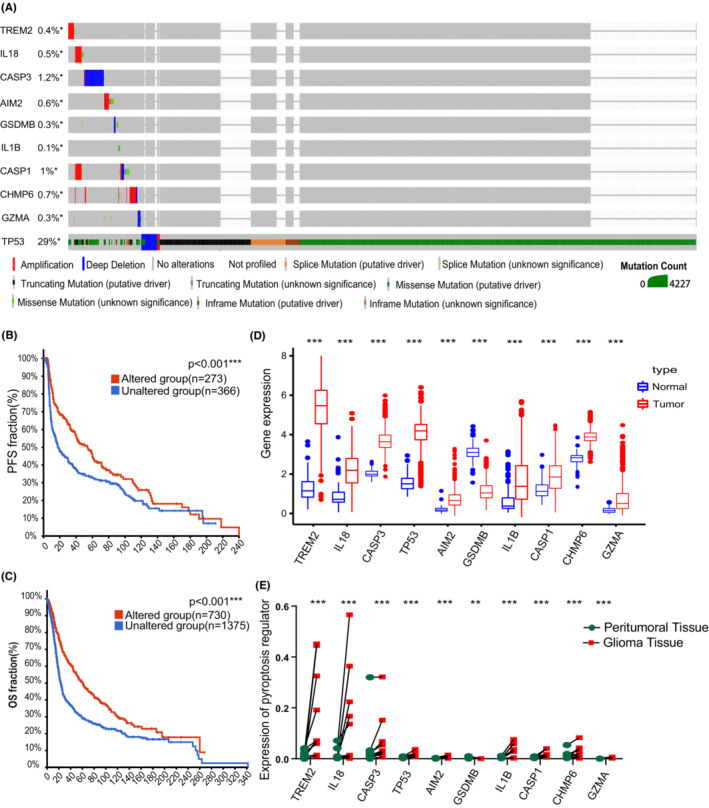
Characteristics and differences of pyroptosis‐related regulators in gliomas. (A) Landscape of genomic alteration profiles in glioma patients. Corresponding colors had annotations at the bottom which mean different mutation types. (B) Effect of genomic alteration on the disease‐free survival (PFS) of gliomas. (C) Effect of genomic alteration on the overall survival (OS) of gliomas. (D) Expressions of pyroptosis‐related regulators between normal tissues (*n* = 108) and glioma tissues (*n* = 660) in TCGA cohort. (E) Expressions of pyroptosis‐related regulators in 12 pair peritumoral brain tissues and corresponding glioma tissues from Guangdong Provincial People's Hospital. *p < 0.05; **p < 0.01; ***p < 0.001; ns, not statistically significant.

A sum of 42 pyroptosis‐related genes were identified, followed by further analysis of differentially expressed genes (DEGs) in glioma. Consequently, 20 pyroptosis‐related regulators were screened out in the TCGA‐GTEx cohort, whereas 18 pyroptosis‐related regulators showed significant differences between normal brain tissues and glioma tissue in the CGGA‐GTEx cohort (Figure 1A–D). Next, a protein network was used to detect and visualize the ten common DEGs (Figure 1E,F). At the genetic level, *TP53* showed the highest frequency of mutations, whereas *IL1B* showed the least frequency of mutations (Figure 2A). Taking clinical data of gliomas into consideration, alteration of these regulators was correlated to better prognosis of gliomas (Figure 2B,C). Moreover, ten pyroptosis‐related regulators were upregulated in glioma samples compared to normal brain tissues, with exception of *GSDMB* (Figure 2D,E and Figure S1–S3).

### 3.2 Glioma classification pattern on the basis of pyroptosis‐related regulators

**FIGURE 3:**
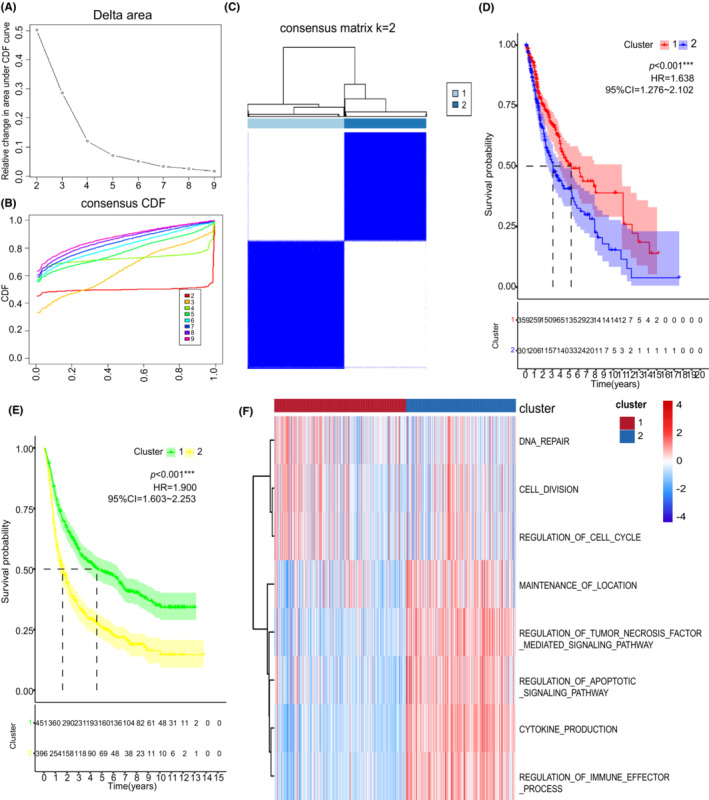
Subgroups of gliomas related by pyroptosis‐related regulators. (A) Cumulative distribution function curve and (B) delta area of immune‐related genes in TCGA cohort. (C) Consensus score matrix of all samples when k = 2 in TCGA cohorts. (D) OS curves for the two pyroptosis‐related clusters based on 660 gliomas from TCGA cohorts. (E) Kaplan–Meier curves showing OS of pyroptosis‐related clusters in CGGA cohort. (F) An aggregate of the potential biological interaction of pyroptosis‐related regulators from STRING platform. The heatmap was used to visualize biological processes analyzed by GSVA which showed the active biological pathways in distinct pyroptosis‐related clusters. ***p < 0.001.

On the basis of ten pyroptosis‐related regulators, the consensus clustering method was performed, and two subtypes were identified: pyroptosis‐related IS1 in 359 samples and pyroptosis‐related IS2 in 301 samples (Figure 3A–C). IS1 glioma patients had better survival prognosis than IS2 patients in both TCGA and CGGA cohorts (Figure 3D,E). The potential biological behaviors associated with these clusters were detected in the STRING platform based on the protein network mentioned above. Furthermore, gene set variation analysis (GSVA) was performed to analyze the biological processes significantly correlated with IS2 and IS1 (Figure 3F). Results showed that IS1 was mainly enriched in pathways associated with carcinogenic activation pathway, such as cell division, DNA repair, and negative regulation of ubiquitination. On the other hand, IS2 was mainly enriched in immune‐related signaling pathways, including cytokine production and regulation of immune effector process.

### 3.3 Differences in clinical characteristics and TME infiltration between the two pyroptosis‐related subtypes

**FIGURE 4:**
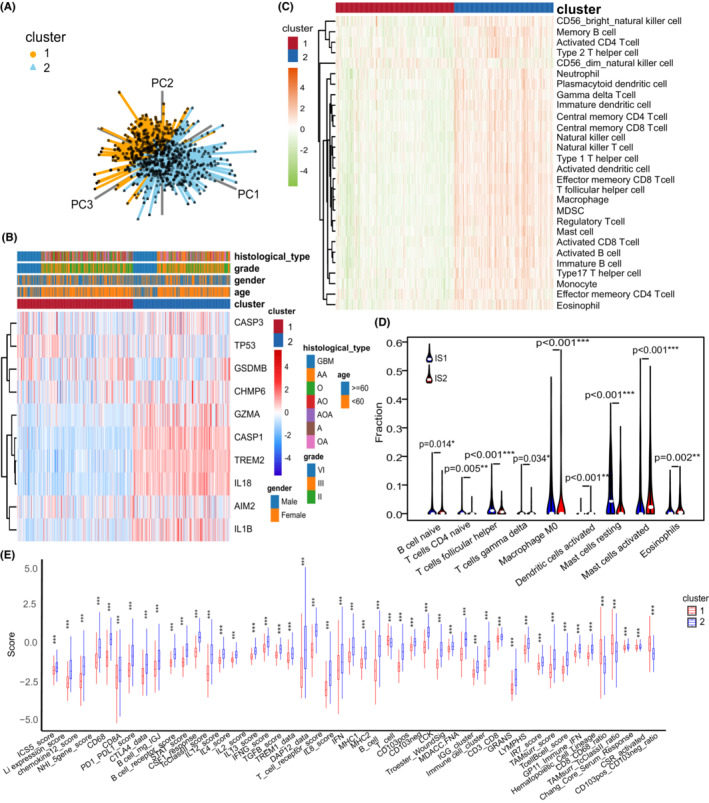
Different pyroptosis‐related clusters showed diverse clinical features and TME cell infiltration. (A) Principal component analysis for the expression of pyroptosis‐related regulators to distinguish IS1 (*n* = 359) from IS2 (*n* = 301) in TCGA cohorts. (B). Consensus clustering of differential expression genes between the two pyroptosis‐related clusters in the TCGA cohort. (C) The abundance of TME infiltrating cells between the two pyroptosis‐related clusters analyzed by 28 previous reported immune signatures in the TCGA cohort. (D) CIBERSORT algorism showed the difference of immune infiltrates in two pyroptosis‐related clusters. € Differential enrichment scores of 56 immune signatures between two pyroptosis‐related clusters. * < 0.05; **p < 0.01; ***p < 0.001; ns, not statistically significant.

The two pyroptosis‐related patterns could be used to distinguish glioma samples (Figure 4A). Therefore, we performed a comprehensive analysis of the association between clinical information and this pyroptosis‐related classification. Interestingly, the expression level of most pyroptosis‐related regulators was higher in cluster 2 than in cluster 1 (Figure 4B). Next, the immune signatures of gliomas were analyzed to evaluate the immune status of different clusters. The two glioma subtypes had distinct characteristics of TME immune cell infiltration where IS2 can be considered to be immunologically “hot” for highly abundant immune cell infiltration, whereas IS1 was defined as an immunologically “cold” phenotype with a sparse population of immune cells (Figure 4C). The CIBERSORT algorithm showed that IS1 was enriched with follicular helper T cells, naive B cell, and resting mast cells, whereas IS2 was enriched with activated mast cells and activated dendritic cells (Figure 4D). Furthermore, most immune signatures in IS2 were higher than those in IS1, which validated our abovementioned finding (Figure 4E). Collectively, these results suggest that pyroptosis‐related patterns with different molecular characteristics can be potential biomarkers for different tumoral immune statuses.

### 3.4 Development of a pyroptosis‐related signature and exploring its biofunction

**FIGURE 5:**
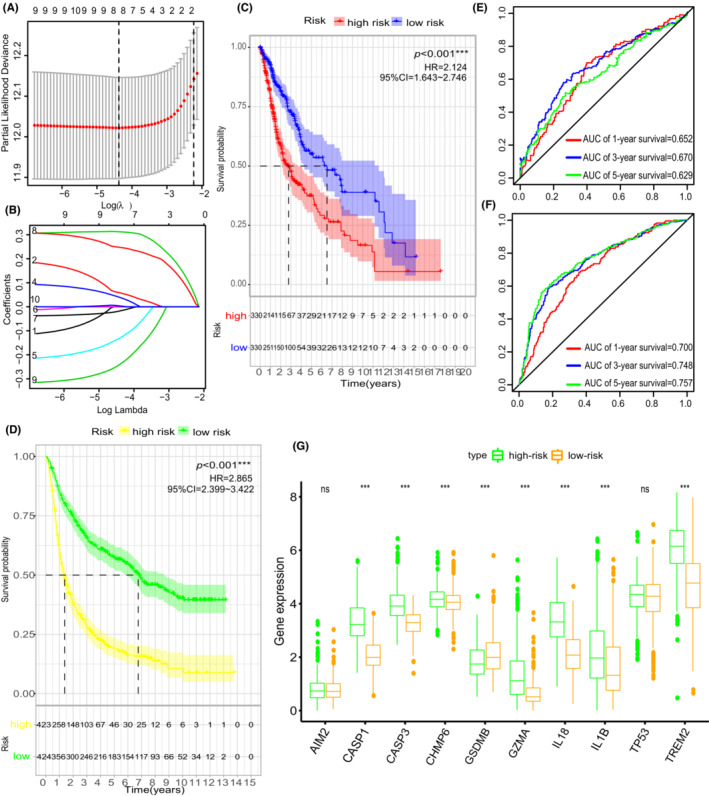
Generation of a gene expression signature to predict patient survival based on pyroptosis‐related clusters. (A, B) In the LASSO‐Cox model of the TCGA cohort, the minimum standard was adopted to obtain the value of the super parameter λ by 10‐fold cross‐validation. (C) OS curves for the different PS‐score subgroups about 660 patients with glioma from the TCGA cohort. (D) OS curves for the different PS‐score subgroups about 847 glioma samples from CGGA cohorts. (E, F). Time‐dependent receiver operating characteristic (ROC) analysis of the PS‐score in the TCGA cohort and CGGA cohort. (G) Expressions of pyroptosis‐related regulators between different PS‐signature groups in the TCGA cohort. *p < 0.05; ** < 0.01; ***p < 0.001; ns, not statistically significant.

To apply the cluster method in glioma evaluation, we developed a gene signature that could predict the diagnosis and treatment of each glioma patient. Three of the 10 pyroptosis‐related regulators (*CASP1*, *CASP3*, and *IL18*) were filtered out in the LASSO‐Cox regression model and used to construct a pyroptosis‐related signature (Figure 5A,B and supplementary Figure 2). Coefficients of the three hub pyroptosis‐related regulators were calculated and listed in table 2. Next, we divided glioma patients into the low‐risk group and high‐risk group to determine the prognosis predicting value of “PS‐score”. Low‐risk group glioma patients had a survival advantage over those in the high‐risk group (Figure 5C,D). Time‐dependent ROC analysis further proved that the PS‐score had a good diagnostic performance in glioma patients (Figure 5E,F). Last but not least, ten pyroptosis‐related regulators were upregulated in high‐risk group compared to low‐risk group, with exception of *GSDMB* (Figure 5G).

**TABLE 2:** Coefficient of pyroptosis‐related regulators

Pyroptosis‐related regulators	Coefficient
*CASP1*	0.214
*IL18*	0.052
*CASP3*	0.322

Abbreviations: *CASP1*, caspase 1; *CASP3*: caspase 3; *IL18*, interleukin 18.

**FIGURE 6:**
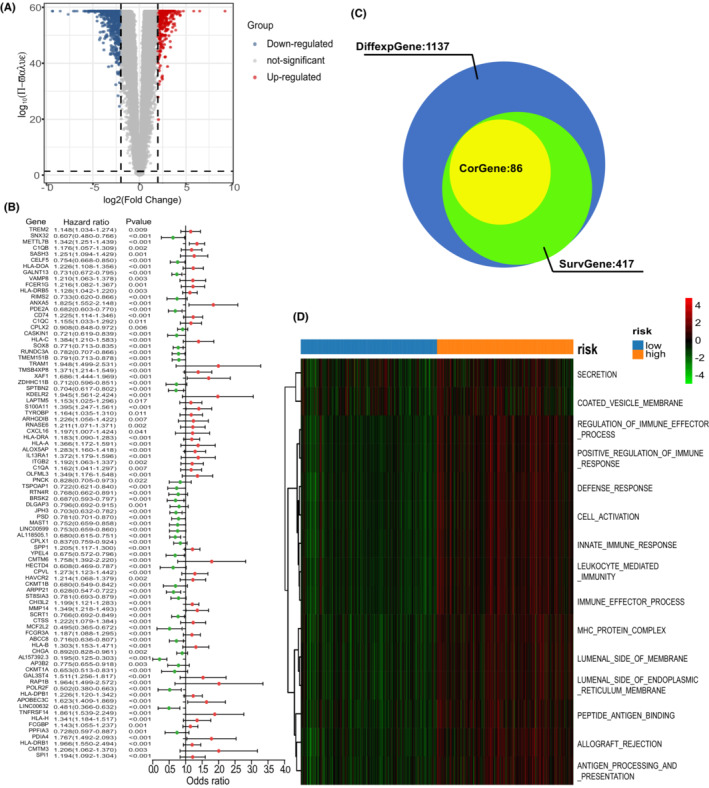
Function enrichment analysis of co‐expressed genes associated with pyroptosis‐related signature (PS‐signature). (A) An overview of the differential gene expression between the gliomas and normal brain tissues in TCGA‐GTEx cohorts. (B) Univariate Cox regression analyses of OS in TCGA cohorts. The *p*‐values were obtained by Univariate Cox regression. (C) Venn plots show the PS‐signature related genes. (D) Heatmap was used to visualize biological processes analyzed by GSEA which showed the active biological pathways in the distinct group of PS‐signature.

The association between risk‐score and clinical factors was also assessed. The risk‐score was at quantitatively higher levels in IS2 than in IS1 (Figure S3A). However, it showed no difference between lower‐grade glioma (LGG, grade II and grade III) and glioblastoma (GBM, grade IV) (Figure S3B). Moreover, patients in the low‐risk score group had better prognostic outcomes than patients in the high‐risk score group in both LGG and GBM (Figure 3C,D). Furthermore, the PS‐score showed significant difference in the grouping of the other clinical factors (histologic type, age, *IDH* status, and 1p19q status) (Figure 3E–H). Given that the prognostic label of glioma plays a significant role in glioma treatment, we explored the function of the PS‐score. In total, 1137 DGEs were detected in the TCGA‐GETx cohort (*p* < 0.05, | log2FC | >2) (Figure 6A). A Spearman correlation analysis was then performed to assess the correlation between PS‐score and prognostic DEGs. Target genes (Spearman's rank correlation analysis | r |>0.5, *p* < 0.001) was selected for further analysis (Figure 6B,C). Finally, functional enrichment analysis based on Gene Set Enrichment Analysis (GSEA) was performed on the detected target genes to determine their biofunction (*p* < 0.05, Figure 6D).

### 3.5 The PS‐score could indicate TME differences

**FIGURE 7:**
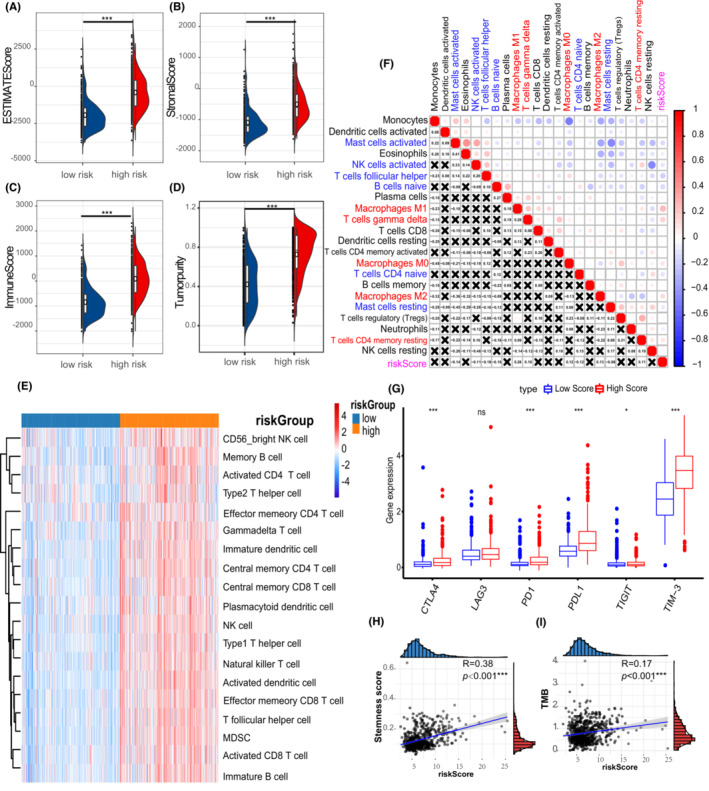
Different PS‐signature showed diverse clinical features and TME cell infiltration. Association between PS‐score and ESTIMATE signature: (A) Estimate score, (B) Stromal score, (C) Immune score, and (D) Tumor purity. (E) Abundance of TME infiltrating cells between different PS‐signature groups by 28 previous reported immune signatures. Columns of the heatmap represented 660 glioma samples. (F) Correlation between TME infiltration and PS‐signatures by CIBERSORT signature. (G) Differential expression of six immune checkpoints between different PS‐signature groups. (H) Correlation analysis between PS‐score and stemness score. (I) Correlation analysis between PS‐score and TMB. *p < 0.05; **p < 0.01; ***p < 0.001; ns, not statistically significant.

Subsequently, the association between immune infiltration and PS‐score was investigated. The ESTIMATE analysis revealed that immune‐related scores were higher in the high PS‐score group than in the low PS‐score group (Figure 7A–D). We found the high PS‐score group showed more abundant immune infiltration than the low PS‐score group, which confirmed the pre‐described result (Figure 7E). Importantly, anti‐tumor immune cells, including activated NK cells and follicular helper T cells, showed negative correlations with PS‐score, whereas the resting memory cells and macrophages had a positive trend with PS‐score (Figure 7F). It is worth noting that immune checkpoints reflect the immunosuppressive status of a tumor. Herein, it was found that the immune checkpoints were upregulated in the samples of the high PS‐score group than those of the low PS‐score group (Figure 7G). Furthermore, the TMB and stemness score of different PS‐scores were calculated. The high PS‐score group had a higher TMB and stemness score than the low PS‐score group (Figure 7H,I), suggesting that the PS‐score was positively correlated with higher glioma malignancy. However, immunotherapy still has potential in high PS‐score glioma patients.

### 3.6 The PS‐score could predict prognosis in clinical scenarios

**FIGURE 8:**
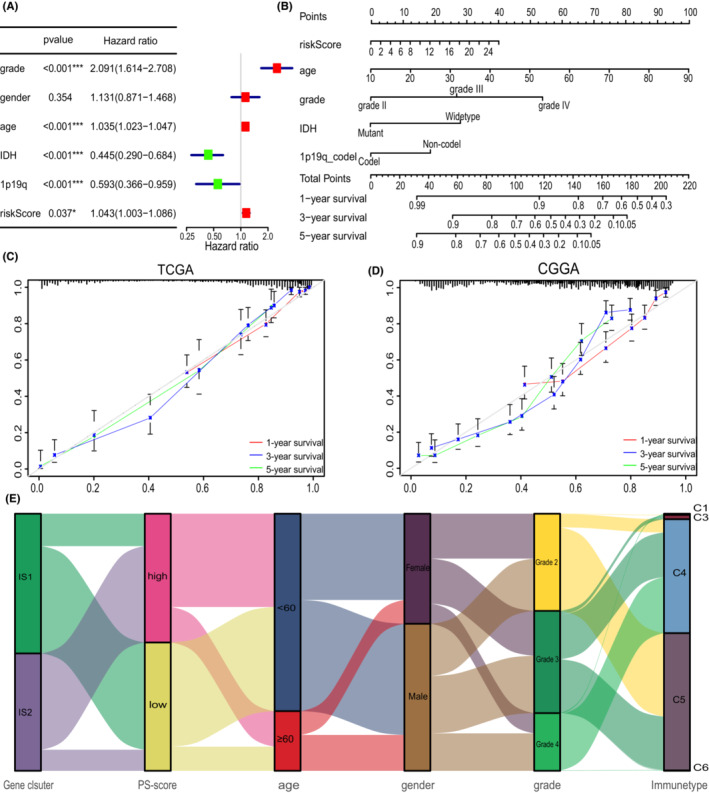
Characteristics of the PS‐signature model. (A) Multivariate Cox regression analysis showing the risk signature as an independent prognostic factor in glioma. (B) Nomogram predicting 1‐, 3‐ and 5‐year OS in the TCGA cohort. (C) Calibration plots of the nomogram predicting OS at 1, 3, and 5 years in the TCGA cohorts. (D) Calibration plots of the nomogram predicting OS at 1, 3, and 5 years in the CGGA cohorts. (E) Alluvial diagram showing the changes of pyroptosis‐related clusters, PS‐score, age, gender, grades, pan‐cancer immune subtypes in TCGA cohort. *p < 0.05; ***p < 0.001.

Furthermore, the risk‐score and other clinicopathologic characteristics were incorporated into multivariate Cox regression analysis. Results showed that the PS‐score can be regarded as an independent prognostic factor of glioma (Figure 8A). Given the high predictive capability of the PS‐score, a nomograph integrating PS‐score and four clinicopathologic factors was constructed with the aim of predicting the survival rates of glioma patients at 1, 3, and 5 years (Figure 8B). The calibrations showed good predictive value for gliomas in both TCGA and CGGA cohorts (Figure 8C,D). Finally, the alluvial diagram displayed interaction of different characteristics in glioma patients (Figure 8E).

## 4 DISCUSSION

Pyrptosis is a special kind of programmed cell death in response to pathogens.^38^ Splicing of an important pyroptosis‐related regulator, *GSDMD*, suppresses several oncogenic pathways (*MAPK*, *PI3K‐mTOR*, and *WNT*).^39^, ^40^, ^41^ It has been reported that cleavage of *GSDMD* can switch apoptosis to pyroptosis, which promotes the function of lymphocytes in breast cancer cells, facilitates tumor necrosis, and predicts poor prognosis in patients.^42^ This suggests that the prognostic value of pyroptosis‐related regulators in glioma cannot be judged based on their performance in other tumors. Therefore, studies should be conducted to further explore the pathways and pyroptosis associated molecules in glioma, with the overarching goal of creating a prognostic signature to help clinicians understand the impact of these genes' expression.

In our study, we detected ten differentially expressed pyroptosis‐related regulators in glioma. Among them, *TREM2* can inhibit *CASP1‐*related pyroptosis and promote host resistance to inflammation.^43^ In glioma, overexpression of *TREM2* enhances tumor cell proliferation and invasion.^44^ *AIM2* takes part in the construction of inflammasomes who recognizes double‐stranded DNA breaks (DSBs) and motivate *CASP1* activation and pyroptosis.^45^, ^46^ In addition, *AIM2* inhibited Gli1 expression through the smoothened homolog (SMO)‐independent pathway and regulated tumor cell proliferation and migration in a Gli1‐dependent manner.^47^ Recurrent gliomas often contain driver mutations in *TP53*, which are distinct from those observed in the initial tumor.^48^ *CASP1* is upregulated by *TP53* in response to stress, thereby inducing cell necrosis and suppressing oncogenic transformation.^49^, ^50^ *CHMP6* was reported to disrupt mitochondrial potential and reduce ATP synthesis, causing cellular swelling and cell death.^51^ However, *CHMP6* also interacts with Ras to harbor it on the endosome, playing a significant role in EGFR recycling and enhancing growth factor signaling.^52^ Furthermore, the expression of these pyroptosis‐related regulators showed significant difference in two subtypes due to various heterogeneities. Interestingly, IS2 had more infiltration of immune cells than IS1, but it also had a worse prognosis. It was previously reported that inflammation and necrosis might promote the migration and invasion of glioma stem cells (GSCs).^53^ Therefore, we speculated that the complicated microenvironment caused by pyroptosis leads to genetic alteration and upregulation of oncogenes, thereby stimulating tumor proliferation.

To accomplish better clinical application of the PS‐related cluster, a PS‐related signature was constructed for predicting glioma prognosis based on pre‐identified subtypes. Through Lasso regression analysis, three pyroptosis‐related regulators (*CASP1*, *CASP3*, and *IL18*) was identified to construct the model. A previous study reported that *CASP1* assists in regulating T cell immunity and innate immunity in three pyroptosis‐related regulators of PS‐related signature, which might indicate their important roles in tumor checkpoint inhibition.^54^ IL‐18 was also shown to elicit anti‐glioma response in vivo through production of IFN‐γ and NO from macrophages and NK cells.^55^ However, another study reported that microglia secrete IL‐18 to promote migration of glioma in the tumor microenvironment.^56^ Although the high PS‐score group showed abundant infiltration of immune cells, more immunosuppressive APCs and regulatory T cells were also localized in these samples, suggesting that pyroptosis in glioma indeed invoked more immune response. According to a previous study, patients with higher somatic TMB exhibited enhanced responses, lasting clinical benefits, and long‐term survival after treatment with immune checkpoint blockade therapy.^36^ Fortunately, a high‐risk score was positively correlated with TMB, providing hope for immunotherapy. In brief, the PS‐signature was not only significantly associated with glioma prognosis, but it also had important reference value for immunotherapy.

In this study, we perform a comprehensive analysis of pyroptosis‐related regulators and construct a pyroptosis‐related model to predict glioma prognosis. Actually, Gao et al have shown that increasing expression levels of GSDMD were associated with aggressiveness of NSCLC, including higher TNM stage and larger tumor volume.^57^ Suppression of GSDMD inhibited the activation of EGFR/Akt signal in cancer cells and impede their proliferation. Therefore, this finding suggested that some of pyroptosis regulators play cancer‐promoting effect in glioma, which is consistent with the previous study. Chemotherapy and radiotherapy were important inducers of glioma pyroptosis for tumor necrosis and following immune response.^58^ Nevertheless, *CASP3* was reported to activate iPLA2 and stimulate tumor cell repopulation after radiotherapy, increasing recurrence rate and deaths of patients.^59^ Besides, hypoxia is one of the most important factors in the tumor micro‐environment because it modulates the anti‐tumor immune response and is associated with tumor radio‐resistance. Hypoxia‐induced formation of the PD‐L1/STAT3 complex promotes expression of *GSDMC* to induce pyroptosis, followed by tumor necrosis in hypoxic regions, which suppress antitumor immune response from pyroptosis and is critical to tumor proliferation.^42^, ^60^ In conclusion, our findings of the unexpected effects of pyroptosis in glioma are intriguing and warrant further investigation.

## 5 CONCLUSION

Glioma exhibits complex immune microenvironment, such as immunosuppression of infiltrating macrophages and exhaustion of T cells. As an important immune response, pyroptosis plays significant roles in tumor proliferation, invasion, and metastasis. Our findings provide a novel insight on the biofunction of pyroptosis in glioma. Herein, a pyroptosis‐related subtyping and a PS‐signature of glioma was constructed for predicting the prognosis of glioma and reflecting the immune status of patients.

## AUTHOR CONTRIBUTIONS

S.W.C. and S.J.Z. designed the study, checked the data, and prepared the manuscript. J.C. and S.W.C. performed data collection, searched the literature, and took part in the manuscript preparation. J.C. and B.X.L conducted the statistical analysis. S.J.Z. and H.L. supervised this project. All authors read and approved the final manuscript.

## FUNDING INFORMATION

This program was financially supported by the Natural Science Foundation of China (NO.81901250), High‐level Hospital Construction Project of Guangdong Province of China (NO. DFJH201924), GDPH Scientific Research Funds for Leading Medical Talents and Distinguished Young Scholars in Guangdong Province (NO. KJ012019434), and the Natural Science Foundation of Guangdong Province of China (NO.2018A0303130236).

## CONFLICT OF INTEREST

The authors declare that the research was conducted in the absence of any commercial or financial relationships that could be construed as a potential conflict of interest.

## ETHICS APPROVAL AND INFORMED CONSENT

The study was approved by the Research Ethics Committee of Guangdong Provincial People's hospital, Guangdong Academy of Medical Science (No. GDREC20190145H[R2]). All patients provided written informed consent.

## Supporting information


Figure S1–S3

## Data Availability

The datasets analyzed during the current study are available in The Cancer Genome Atlas database (TCGA, https://portal.gdc.cancer.gov/), Chinese Glioma Genome Atlas (CGGA, http://www.cgga.org.cn/), and Geneotype‐Tissue Expression database (GTEx, https://www.gtexportal.org/).
