# Supplementary material for: A pyroptosis‐related signature predicts prognosis and indicates immune microenvironment infiltration in glioma
Source: Cancer Med. 2022 Sep 26;12(4):5071–87. doi: 10.1002/cam4.5247 (PMC9972150; doi:10.1002/cam4.5247)
Supplement: Supplementary file 1 — Figure S1–S3 [file CAM4-12-5071-s001.docx]

Supplementary Material


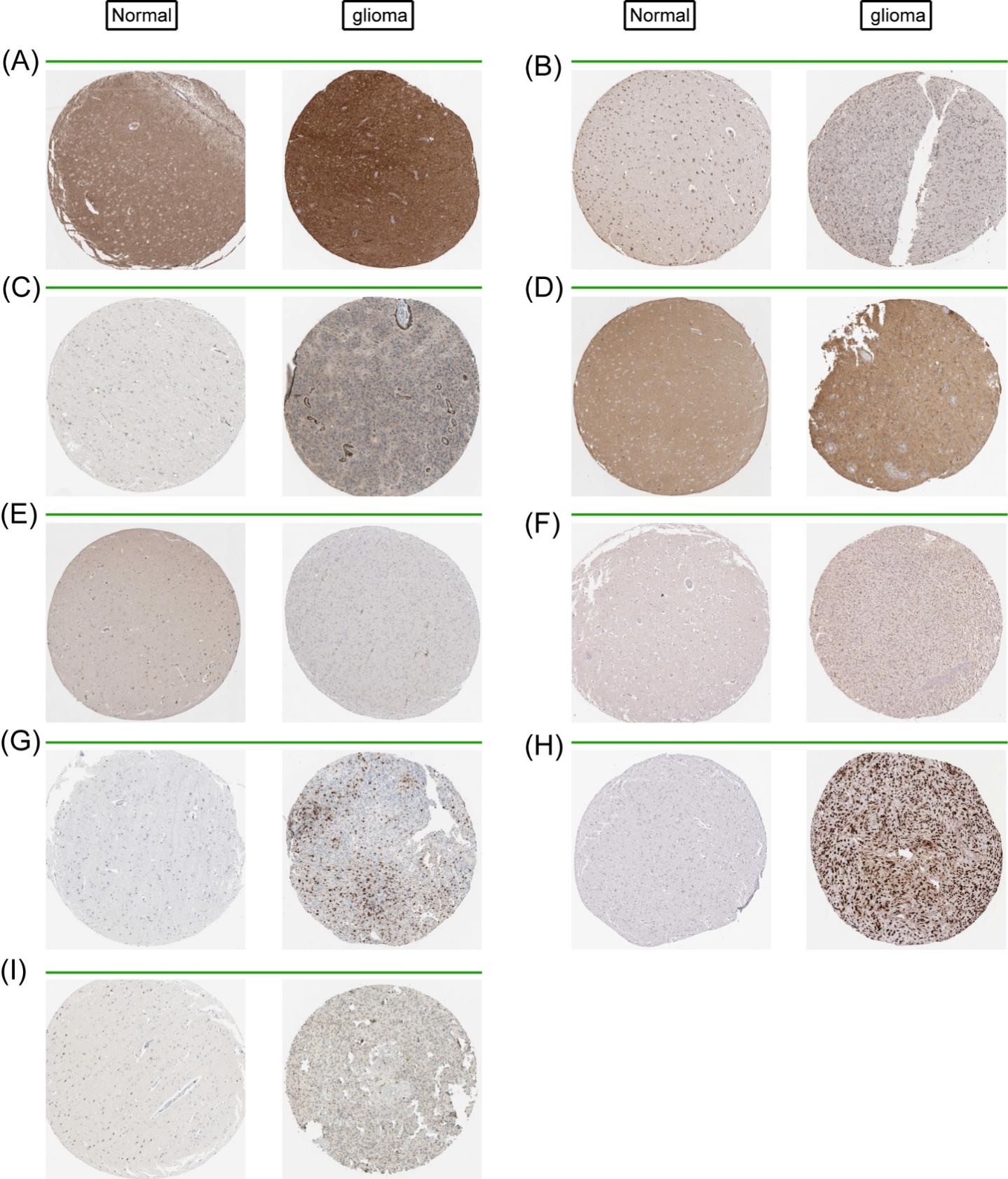


Supplementary Figure 1. Immunochemistry staining results from HPA database were used to detect the expression of pyroptosis-associated regulators. (A) Differential expression of *AIM2* between normal brain tissue and glioma samples. (B) Differential expression of *CASP1* between normal brain tissue and glioma samples. (C) Differential expression of *CASP3* between normal brain tissue and glioma samples. (D) Differential expression of *CHMP6* between normal brain tissue and glioma samples. (E) Differential expression of *GSDMB* between normal brain tissue and glioma samples. (F) Differential expression of *GZMA* between normal brain tissue and glioma samples. (G) Differential expression of *IL18* between normal brain tissue and glioma samples. (H) Differential expression of *TP53* between normal brain tissue and glioma samples. (I) Differential expression of *TREM2* between normal brain tissue and glioma samples.


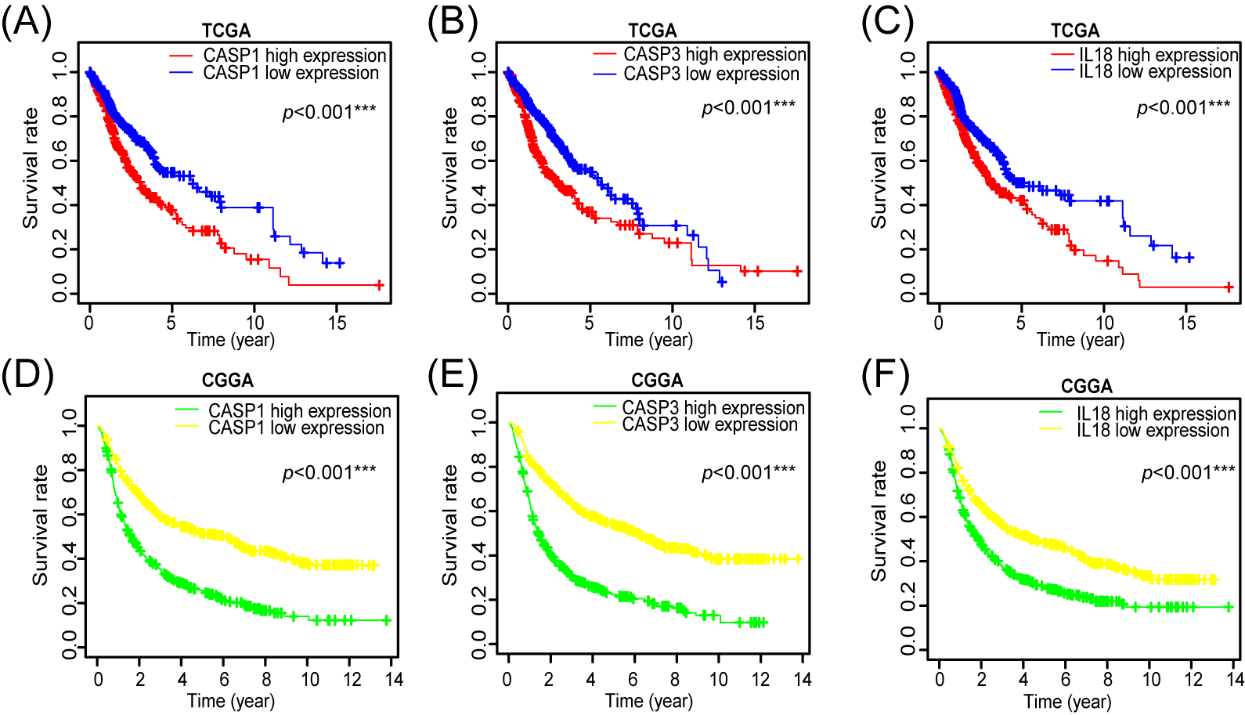


**Supplementary Figure 2.** Prognostic value of three components of PS-signature in glioma. (A) Effect of *CASP1* on glioma patients in TCGA cohort. (B) Effect of *CASP3* on glioma patients in TCGA cohort. (C) Effect of *IL18* on glioma patients in TCGA cohort. (D) Effect of *CASP1* on glioma patients in CGGA cohort. (E) Effect of *CASP3* on glioma patients in CGGA cohort. (F) Effect of *IL18* on glioma patients in CGGA cohort. ***, p<0.001.


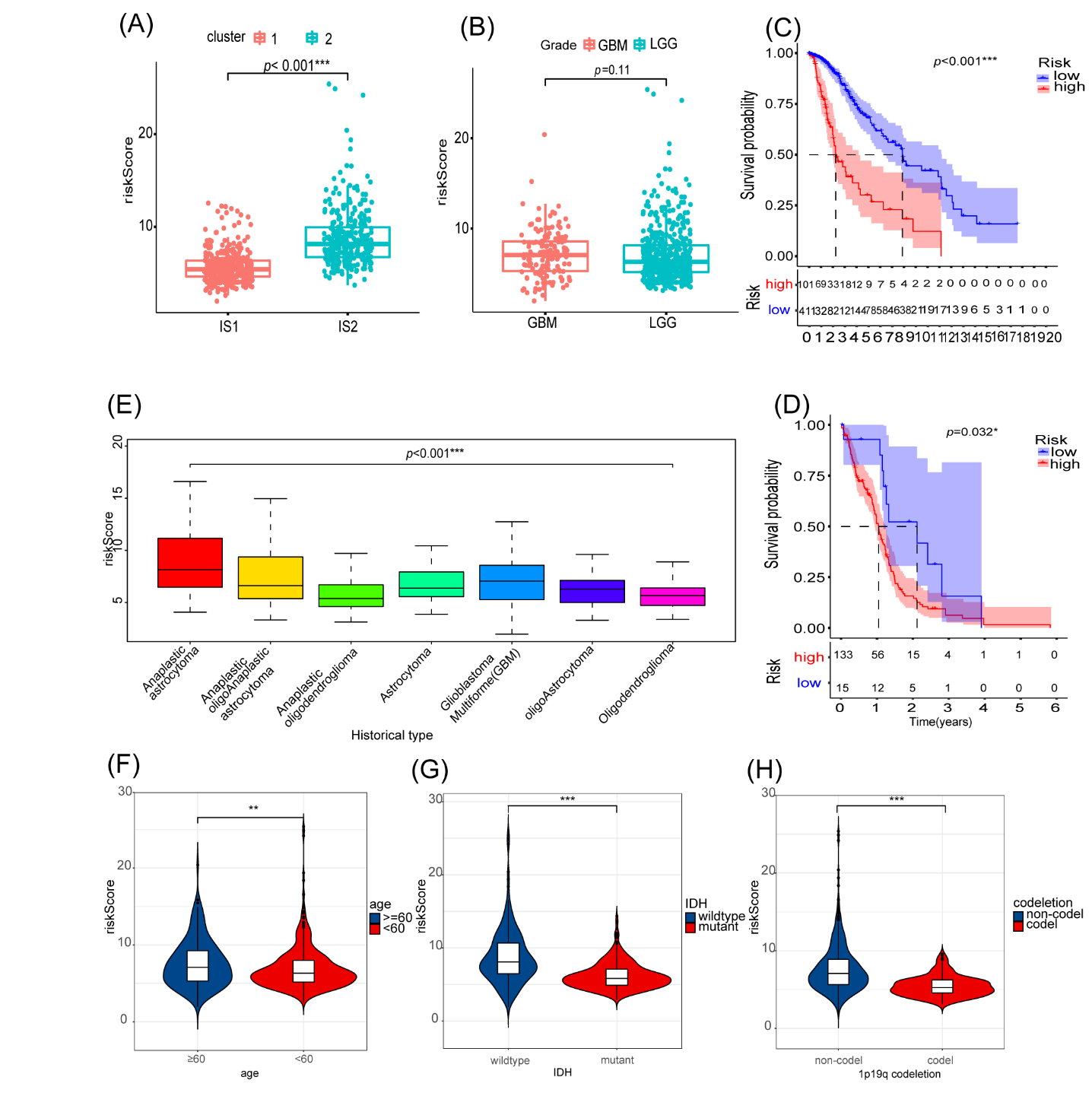


**Supplementary Figure 3.** Prognostic value and clinical characteristics of PS-signature in glioma. (A) Association between risk signature and pyroptosis related clusters of gliomas in TCGA cohort. (B) Association between risk signature and grades of gliomas in TCGA cohort. (C) The prognostic value of PS-signature in LGG of TCGA cohort. (D) The prognostic value of PS-signature in GBM of TCGA cohort. (E) Association between risk signature and histological types of gliomas in TCGA cohort. (F) Association between risk signature and age of gliomas in TCGA cohort. (G) Association between risk signature and isocitrate dehydrogenase (*IDH*) status of gliomas in TCGA cohort. (H) Association between risk signature and 1p19q status of gliomas in TCGA cohort. *, p<0.05; **, p<0.01; ***, p<0.001.
